# Differential Modulation of TCF/LEF-1 Activity by the Soluble LRP6-ICD

**DOI:** 10.1371/journal.pone.0011821

**Published:** 2010-07-28

**Authors:** Brandon Beagle, Gail V. W. Johnson

**Affiliations:** 1 Departments of Anesthesiology and Pharmacology and Physiology, University of Rochester, Rochester, New York, United States of America; 2 Department of Cell Biology, University of Alabama at Birmingham, Birmingham, Alabama, United States of America; Ohio State University, United States of America

## Abstract

The canonical Wnt/β-catenin (Wnt) pathway is a master transcriptional regulatory signaling pathway that controls numerous biological processes including proliferation and differentiation. As such, transcriptional activity of the Wnt pathway is tightly regulated and/or modulated by numerous proteins at the level of the membrane, cytosol and/or nucleus. In the nucleus, transcription of Wnt target genes by TCF/LEF-1 is repressed by the long Groucho*/*TLE co-repressor family. However, a truncated member of the Groucho/TLE family, amino terminal enhancer of Split (AES) can positively modulate TCF/LEF-1 activity by antagonizing long Groucho/TLE members in a dominant negative manner. We have previously shown the soluble intracellular domain of the LRP6 receptor, a receptor required for activation of the Wnt pathway, can positively regulate transcriptional activity within the Wnt pathway. In the current study, we show the soluble LRP6 intracellular domain (LRP6-ICD) can also translocate to the nucleus in CHO and HEK 293T cells and in contrast to cytosolic LRP6-ICD; nuclear LRP6-ICD represses TCF/LEF-1 activity. In agreement with previous reports, we show AES enhances TCF/LEF-1 mediated reporter transcription and further we demonstrate that AES activity is spatially regulated in HEK 293T cells. LRP6-ICD interacts with AES exclusively in the nucleus and represses AES mediated TCF/LEF-1 reporter transcription. These results suggest that LRP6-ICD can differentially modulate Wnt pathway transcriptional activity depending upon its subcellular localization and differential protein-protein interactions.

## Introduction

The canonical Wnt/β-catenin (Wnt) pathway is a ubiquitous transcriptional regulatory pathway that affects the expression of over 1800 genes involved in at least 36 different pathways [1], [2], [3], [4], [5]. As a result, the Wnt pathway influences a diversity of biochemical functions including cell patterning, differentiation, embryonic development and apoptosis [1], [2], [3], [5]. Defects at various levels of the pathway therefore result in numerous pathological conditions including various cancers and neurodegenerative disorders [3], [6].

In the absence of Wnt pathway activation, cytosolic β-catenin (a co-activator for TCF/LEF-1) is phosphorylated by GSK3β and CKIα [2], [3], [4], [7]. Phosphorylation promotes β-catenin degradation resulting in low basal levels of cytosolic β-catenin thus preventing β-catenin nuclear translocation and activation of the Wnt responsive transcription factor TCF/LEF-1 [2], [3], [8], [9], [10]. The TCF/LEF-1 family (TCF-1,-3, -4 and LEF-1) mediates expression of Wnt target genes by acting as a repressor or activator of Wnt target genes [9], [10]. In the absence of significant nuclear β-catenin, DNA bound TCF/LEF-1 proteins repress expression of Wnt target genes by interacting with the long Groucho/TLE family of co-repressors [8], [9], [10], [11], [12], [13], [14]. Long Groucho/TLE's are pentadomain proteins composed of highly conserved amino-terminal Q domain (protein interaction and repression), followed by a GP domain (repression), CcN domain (nuclear localization), an SP (repression) and highly conserved WD40 domain (protein interaction) [10], [11], [15]. The Q domain mediates interaction with transcription factors such as TCF/LEF-1 as well as tetramerization of Groucho/TLE members, which is essential for its repressor function and interaction with TCF/LEF-1 [8], [10], [11], [12], [14], [15], [16], [17], [18], [19], [20]. The GP domain also has a critical repressor function as it is essential for interaction between long Groucho/TLE's and co-repressor histone deacetylases (HDACs) [8], [10], [11], [15], [16], [20], [21].

In addition to the full-length Groucho/TLE members, there is also a shorter member called amino-terminal enhancer of split (AES) that contains only the Q and GP domains [10], [11], [12], [15], [17], [18], [20], [22], [23]. *AES* is not an alternatively spliced variant of the long *Groucho/TLE* gene but is a distinct family member constitutively expressed from its own loci [10], [18]. The Q domain of AES and the long Groucho/TLE members both mediate multimerization between AES and/or long Groucho/TLE proteins [8], [10], [11], [15], [17], [18], [20], [24] as well as interactions with TCF/LEF-1 proteins [8], [10], [12], [14]. However, the GP domain of AES and long Groucho/TLE members are conserved but functionally distinct, as AES does not interact with transcriptionally repressive HDAC proteins [10], [11], [15], [18], [25]. Because AES multimerizes with long Groucho/TLE members but does not interact with HDAC's, long Groucho/TLE proteins lose their ability to form functional di/tetramers and/or bind transcription factors [10], [11], [15], [18]. As a result, AES can antagonize the repressor function of long Groucho/TLE members [10], [11], [12], [15], [17], [18], a potential mechanism by which AES positively modulates TCF/LEF-1 activity [10], [12]. Interestingly, AES can function as a co-activator or co-repressor of various transcription factors, which also distinguishes it from the dedicated repressor function of long Groucho/TLE members [11], [15], [21]. AES is also highly conserved among species as xenopus (NP_001083532) and murine (NP_034477.1) homologs share ∼90% and 99% protein sequence identity to human AES (NP_001121.2).

Activation of the Wnt pathway requires interaction between the secreted Wnt protein and two cell surface receptors, Frizzled (Fz) and LRP6 (Low density lipoprotein receptor Related Protein 6)(the closely related LRP5 can also act as a co-receptor for certain physiological processes) [2], [3]. The ternary Wnt-Fz-LRP6 complex inhibits cytosolic β-catenin phosphorylation/degradation, resulting in its cytosolic accumulation and translocation into the nucleus [2], [3], [5], [8], [10], [12], [26]. Nuclear β-catenin directly interacts with TCF/LEF-1 thereby displacing Groucho/TLE, which converts TCF/LEF-1 from a transcriptional repressor to an activator of Wnt target genes [3], [5], [8], [9], [10], [12], [21]. In addition to functioning at the membrane as a primary activator of the Wnt pathway [2], [4], our group has shown exogenous LRP6 undergoes regulated intramembranous proteolysis (RIP) resulting in the release and formation of a soluble LRP6 intracellular domain (LRP6-ICD) [27].

While β-catenin mediated activation of TCF/LEF-1 requires robust activation of the Wnt pathway [9], the transcriptional activity of the Wnt pathway (i.e., β-catenin stabilization and/or TCF/LEF-1 activity) can be modulated by various effector proteins at the level of the membrane, cytosol and/or nucleus [5], [6], [10], [26], [28], [29], [30], [31], [32], [33], [34], [35], [36]. We and others previously have shown the LRP6-ICD can function as an effector of the Wnt pathway by attenuating GSK3β mediated β-catenin phosphorylation thereby enhancing cytosolic β-catenin stabilization [35], [36], [37], [38], [39]. LRP6-ICD expression also enhances TCF/LEF-1 activity [35], [36], [38], which may be an indirect result of enhanced β-catenin stabilization. However, the hypothesized mechanism has yet to be confirmed, which is one aim of the current study. It should also be noted that the LRP6-ICD is a GSK3β substrate, but it does not have to be phosphorylated by GSK3β to interact with [35], [38] and attenuate GSK3β activity [35]. It has been shown that the soluble intracellular domain of various cell surface receptors, such as Fz and LRP1, can translocate to the nucleus and directly modulate gene expression [40], [41], [42], [43], [44]. While the cytosolic function of LRP6-ICD has been studied [35], [36], [37], [38], [39], it is unknown if LRP6-ICD can also translocate to the nucleus and directly modulate TCF/LEF-1 activity, which is the primary focus of the study.

Previously we reported that AES and LRP6-ICD interacted in a yeast 2 hybrid assay [39] but the interaction was not confirmed *in situ*. In the current study, we show the LRP6-ICD can translocate to the nucleus where it interacts with AES. In contrast to cytosolic LRP6-ICD, we show that nuclear localized LRP6-ICD represses transcription of a TCF/LEF-1 reporter and depresses AES mediated activation of the reporter. The current findings suggest LRP6-ICD can differentially modulate Wnt pathway transcriptional activity (i.e., β-catenin stabilization and/or TCF/LEF-1 activity) based upon its subcellular localization and protein-protein interactions.

## Materials and Methods

### DNA Constructs

**Figure 1:**
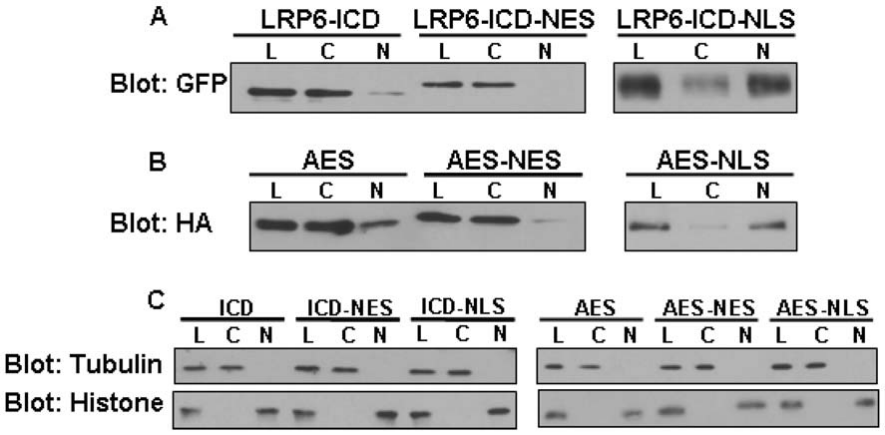
Expression and Subcellular localization of LRP6-ICD and AES constructs. Representative immunoblot of cell lysates from HEK 293T cells which were transiently transfected with LRP6-ICD and AES constructs. Twenty-four hrs post-transfection cells were separated into cytosolic and nuclear fraction and immunoblotted. Lysate (L), cytosolic (C) and nuclear (N) fractions. NES: nuclear export signal. NLS: nuclear localization signal. (A) GFP tagged LRP6-ICD constructs with or without an “NES” or “NLS” tag were detected with an anti-GFP antibody. (B) AES-HA (no localization signal) and AES-HA-NES or AES-myc-NLS constructs were detected with an anti-HA or anti-myc antibody. (C) Histone (nuclear marker) and α-tubulin (cytosolic marker) for the non-fractionated and fractionated lysates shows the relative purity of the fractions.

The GFP tagged wild type LRP6-ICD and LRP6-ICD×5 m (Ser or Thr in all five PPP(S/T)P motifs were mutated to Ala and this construct is not phosphorylated by GSK3β) were described previously [35], [39]. To make the GFP tagged LRP6-ICD constructs with a nuclear localization signal (NLS) or nuclear export signal (NES), wild type GFP-LRP6-ICD or GFP-LRP6-ICD×5 m were used as templates for PCR amplification. The eGFP sequence from the pEGFP-C1 vector (BD Bioscience) was amplified as part of the LRP6-ICD PCR product using the following PCR primers: *SalI-*forward (5′- ACG-CGT-CGA-CCG-CCA-CCA-TGG-TGA-GCA-AGG-GCG-AGG-AG-3′), *BamHI-*forward (5′-CGC-GGA-TCC-CGC-CAC-CAT-GGT-GAG-CAA-GGG-CGA-GGA-G-3′), *NotI-*reverse for wild type GFP-LRP6-ICD (5′-ATA-AGA-ATG-CGG-CCG-CTC-CGG-AGG-AGT-CTG-TAC-AGG-GAG-AG-3′) and *NotI-*reverse for GFP-LRP6-ICD×5 m (5′-ATA-AGA-ATG-CGG-CCG-CTC-CGG-AGG-AGT-CTG-TAC-AGG-GAG-CGG-GTG-GCG-GTG-3′). The amplified GFP-LRP6-ICD products were digested with and subcloned into the *SalI and NotI* site of the pCMV/*myc*/nuc empty vector (EV-NLS; Invitrogen) or *BamHI* and *NotI* site of a pcDNA-NES empty vector to make GFP-LRP6-ICD-myc-NLS or GFP-LRP6-ICD-NES constructs. The pCMV/myc/nuc/GFP vector (EV-GFP-NLS; Invitrogen) was used only as a control for assays involving GFP-LRP6-ICD-NLS constructs. Using human AES cDNA as a template (accession # NM_001130, 197 amino acids), the following PCR primers, digestion and vector were used to make the AES-HA construct: *EcoRI-*forward (5′-GCC-GGG-ATC-CAC-ATG-ATG-TTT-CCA-CAA-AGC-AG-3′) and *EcoRI-*reverse (5′-CCG-GCC-TCG-AGC-TAA-TCC-GAC-TTC-TCG-CCA-TC-3′), digested with and subcloned into the *EcoRI* site of an HA expression vector [35]. To make the AES-myc-NLS or AES-HA-NES constructs using AES-HA as a template, the following PCR primers, digestion and vectors were used for each: *NcoI-*forward (5′-CAT-GCC-ATG-GCA-TGA-TGA-TGT-TTC-CAC-AAA-GCA-GGC-ATT-C-3′) and *XhoI-*reverse (5′-CCG-CTC-GAG-CGG-TCC-ATC-CGA-CTT-CTC-GCC-ATC-3′), digested with and subcloned into the *NcoI* and *XhoI* site of the pCMV/*myc*/nuc vector; *BamHI-*forward (5′-CGC-GGA-TCC-ATG-ATG-TTT-CCA-CAA-AGC-AGG-CAT-TC-3′) and *NotI-*reverse (5′-ATA-AGA-ATG-CGG-CCG-CAA-TGC-GTA-ATC-TGG-AAC-ATC-GTA-TGG-G-3′), digested with and subcloned into the *BamHI* and *NotI* site of the pcDNA-NES vector. The pcDNA-NES vector (from Soner Gundemir, unpublished data) contains the NES of PKI alpha in a pcDNA3.1(+) (Invitrogen) backbone. The integrity of all constructs was confirmed by sequence analysis. Cytosolic and nuclear localization of the NES, NLS and untagged constructs was confirmed by subcellular fractionation (Fig. 1).

### Cell Culture and Transient Transfections

CHO and HEK 293T cells (ATCC) were maintained in Dulbecco's Modified Eagle medium containing 10% FBS. All transient transfections were done using FuGENE 6 transfection reagent (Roche Applied Science) according to the manufacturer's protocol. The final amount of cDNA transfected into the cells was always equalized by using a control β-galactosidase (LacZ) or eGFP unless otherwise noted.

### Luciferase Assay

One day before transfection, 6.0×10^4^ HEK 293T cells were plated in a 12-well plate. Cells were transfected 24 hrs later with 0.2 µg LEF-1 (from Dr. R Grosschedl), 0.005 µg TK-*Renilla* internal control (Promega) and 0.2 µg TOPflash-luciferase reporter (TOP; Upstate) or the negative control reporter, Super8XFOPflash (FOP; Addgene), whose TCF/LEF-1 binding sites have been mutated. Additionally, cells were transfected with AES-HA (0.5 µg), AES-myc-NLS (0.5 µg), AES-HA-NES (0.25 µg), GFP-LRP6-ICD (1 µg), GFP-LRP6-ICD-myc-NLS (1 µg) and/or GFP-LRP6-ICD-NES (0.25 µg). The empty NLS vectors (EV-NLS or EV-GFP-NLS) significantly activated the TOP reporter compared to lacZ or eGFP while the empty pcDNA-NES also had a slight activating effect on the TOP reporter. In contrast to the empty NLS vectors, the activating effect of pcDNA-NES was mitigated by using a lower cDNA concentration. Neither the empty NLS nor NES vectors affected the FOP reporter. However, to mitigate possible confounding variables, the NLS or NES tagged AES-HA and GFP-LRP6-ICD constructs are expressed as fold change relative to the empty NLS or NES vectors. Equal amount of cDNA for the empty NLS or NES vectors and the NLS or NES tagged AES-HA and/or GFP-LRP6-ICD constructs was used in all assays. All experiments were performed at least three times and each time the measurements were done in triplicate.

### Immunblotting, β-catenin assay and Antibodies

Proteins samples were electrophoresed on 10% SDS-polyacrylamide gels (SDS-PAGE), transferred to nitrocellulose membranes and immunoblotted as previously described [35], [39]. Determination of endogenous total cellular β-catenin or cytosolic phospho-β-catenin and total cytosolic β-catenin were carried out using lysis buffer (10 mM Tris, pH 7.5, 10 mM NaCl, 3 mM MgCl_2_, 0.05% Nonidet P-40, 1 mM EGTA with phosphatase and protease inhibitors) or non-detergent lysis buffer as previously described [2], [35], [36]. The following primary antibodies were used: polyclonal anti-phospho-β-catenin (Ser 33/37, Thr 41, Cell Signaling), monoclonal anti-β-catenin (BD Biosciences), monoclonal anti-GFP (Roche), monoclonal anti-HA (Sigma), monoclonal anti-histoneX (Chemicon), monoclonal anti-myc (Cell Signaling), and monoclonal anti-α-tubulin (Sigma).

### Nuclear Fractionation

Separation of cytosolic and nuclear fractionation was carried as previously described [45]. Briefly, cells were washed twice with and harvested in ice-cold PBS and a small aliquot of the total lysate was collected before centrifugation and resuspended in lysis buffer (represents whole cell lysate). The remaining lysate was spun at 800 x *g* for 5 min at 4°C and the cell pellet was resuspended in lysis buffer by triturating followed by centrifugation at 380 × g for 5 min at 4°C. The supernatants were collected and used as the cytosolic fractions. The pellets were washed once in lysis buffer and twice in wash buffer (30 mM sucrose, 10 mM Pipes, pH 6.8, 3 mM MgCl_2_, 1 mM EGTA, 25 mM NaCl with phosphatase and protease inhibitors). The crude nuclei were overlaid on top of 1 m sucrose and spun at 1200 × g for 10 min at 4°C. The pellets were collected and resuspended in buffer B (300 mM sucrose, 10 mM Pipes, pH 6.8, 3 mM MgCl_2_, 1 mM EGTA, 25 mM NaCl, 0.5% Triton X-100 with phosphatase and protease inhibitors) and used as the nuclear fractions.

### Immunoprecipitation

HEK 293T cells were co-transfected with 4 µg of AES-HA along with 5 µg of eGFP-C1 (eGFP), wild type GFP-LRP6-ICD or GFP-LRP6-ICD×5 m. Forty-eight hours post transfection, the AES-HA/eGFP cells were collected with immunoprecipitation buffer (0.5% Nonidet P-40, 150 mM NaCl, 10 mM Tris-Cl (pH 7.4), 1 mM EGTA and 1 mM EDTA with phosphatase and protease inhibitors) while the AES-HA/GFP-LRP6-ICD cell's were fractionated into cytosolic and nuclear fractions. For immunoprecipitation, cytosolic or nuclear fractions (50 µg) were incubated at 4°C for 3 hrs on a rotational shaker with M-280 sheep anti-mouse magnetic IgG beads (Invitrogen/Dynal Biotech) preconjugated to a monoclonal anti-HA antibody (Sigma) in the presence of immunoprecipitation buffer. Cytosolic or nuclear precipitates (and their respective controls) were washed 6× with PBS containing 350 mM NaCl (high salt) and 0.2% Triton X-100 or 2×/2× with high salt PBS/regular PBS. The beads were boiled for 10 min in 2× stop buffer (2% sodium dodecyl sulfate, 250 mM Tris-Cl (pH 7.4), 10% glycerol, 5 mM EDTA and 5 mM EGTA) and subjected to SDS-PAGE and immunoblot analysis.

### Immunofluorescence

Cells were plated onto poly-D-lysine/laminin coated glass coverslips (20/5 µg/ml) and transfected with the indicated constructs for 48 hrs. Cells were fixed with 4% PBS-paraformaldehyde for 15 min, permeabilized with ice cold methanol for 5 min and stained with anti-GFP or anti-HA antibody at 4°C overnight. Cells were then washed with PBS and incubated with 5% FBS containing a Texas red- or FITC -conjugated secondary antibody (Jackson ImmunoResearch) and DAPI (Vectashield, Vector Labs) at room temperature for 1 hr. Slides were analyzed using a Zeiss Axio Observer inverted microscope with a 63× oil immersion objective. Images were captured with an AxioCamMR3 camera (Zeiss Corp) and exported using the AxioVision software (Zeiss Corp.).

### Data Analysis

Data were analyzed using ANOVA between individual groups and considered significantly different when p<0.05. Results were expressed as mean ± S.E.

## Results

### LRP6-ICD translocates to the nucleus

**Figure 2:**
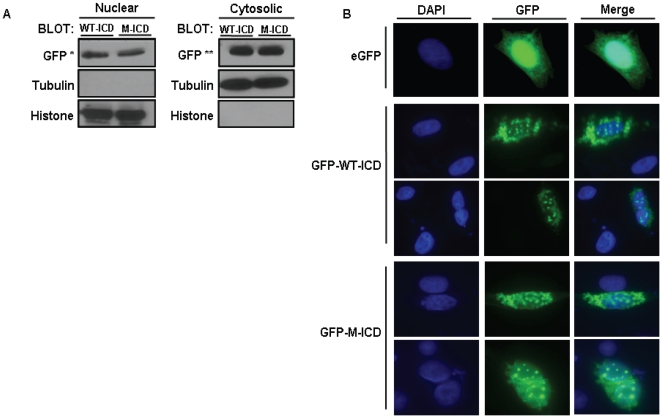
LRP6-ICD localizes to the nucleus and localization is independent of PPP(S/T)P phosphorylation. (A) CHO cells were transfected with wild-type GFP-LRP6-ICD (WT-ICD) or GFP-LRP6-ICD×5 m (M-ICD) for 48 hrs. Nuclear and cytosolic fractions were blotted and sequentially probed for GFP, tubulin (cytosolic marker) and histone (nuclear marker). 20 ug and 5 ug of total nuclear and cytosolic protein were loaded. (*) indicates long exposure and (**) short exposure respectively. (B) CHO cells were transfected with eGFP (empty vector) or wild-type GFP-LRP6-ICD (GFP-WT-ICD) or GFP-LRP6-ICD×5 m (GFP-M-ICD) for 48 hrs. GFP constructs were detected with anti-GFP antibody (middle column; green) and then stained with DAPI to identify nuclei (left column; blue). DAPI and GFP pictures were merged to show nuclear localization of GFP constructs (right column).

It has been shown exogenous LRP6-ICD localizes to the cytosol [36] but nuclear localization of the ∼20 kDa (∼50 kDa with GFP tag) protein has not been examined. Figure 2 clearly shows exogenous wild type LRP6-ICD and a phospho-mutant LRP6-ICD that can not be phosphorylated by GSK3β (LRP6-ICD×5 m) also localize to the nucleus in CHO cells. Although the presence of nuclear LRP6-ICD was evident, the level of nuclear LRP6-ICD relative to cytosolic LRP6-ICD (Fig. 2A), suggests that the majority of LRP6-ICD remains in the cytosol. Immunocytochemistry (Fig. 2B) also show that both wild type LRP6-ICD and LRP6-ICD×5 m localize within DAPI stained nuclei which confirms the fractionation data showing nuclear localization of LRP6-ICD does not require GSK3β phosphorylation. Interestingly, nuclear LRP6-ICD exhibits punctuate nuclear staining whereas cytosolic LRP6-ICD exhibits a more diffuse pattern similar to eGFP. Both LRP6-ICD constructs also exhibited similar nuclear localization in HEK 293T cells (Fig. 1a and Fig. S1). It should also be noted that the subcellular distribution of LRP6-ICD was not significantly altered by co-expression with LEF-1 and/or transcriptional activation of the Wnt pathway by LiCl [7] (data not shown).

### LRP6-ICD differentially modulates TCF/LEF-1 dependent transcription

**Figure 3:**
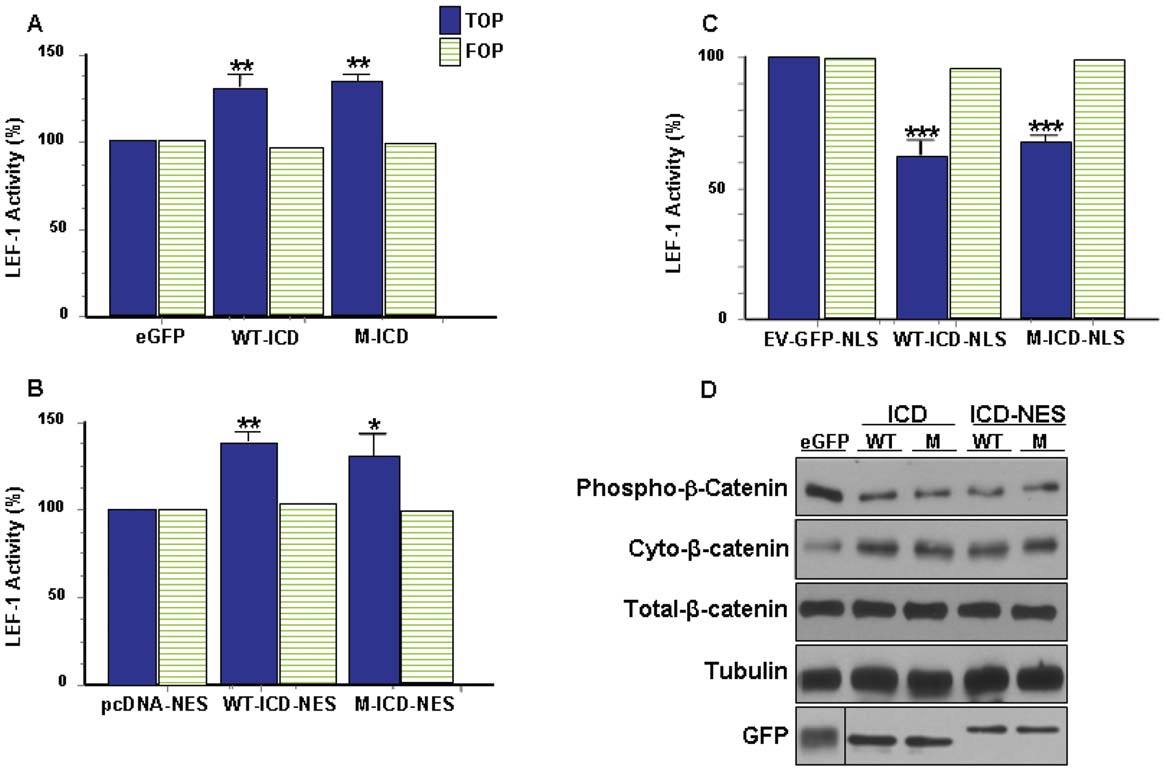
LRP6-ICD differentially impacts TCF/LEF-1 activity in a spatially relevant manner. HEK 293T cells were transfected with luciferase assay reporter constructs along with (A) control eGFP, wild type GFP-LRP6-ICD (WT-ICD) or GFP-LRP6-ICD×5 m (M-ICD) for 24 hrs (B) control pcDNA-NES, wild type GFP-LRP6-ICD-NES (WT-ICD-NES or GFP-LRP6-ICD×5 m-NES (M-ICD-NES) for 48 hrs (C) control EV-GFP-NLS, wild type GFP-LRP6-ICD-NLS (WT-ICD-NLS) or GFP-LRP6-ICD×5 m-NLS (M-ICD-NLS) for 24 hrs and TCF/LEF-1 activity was measured. TCF/LEF-1 activity for the LRP6-ICD constructs is expressed as a percentage of the relevant control used in each condition: (*P<0.01, **P<0.001, ***P<0.0001). (D) HEK 293T cells were transfected with control eGFP, WT-ICD, M-ICD, WT-ICD-NES or M-ICD-NES. Twenty-four hrs post-transfection, cells were collected and cytosolic fractions were probed for endogenous cytosolic phospho-β-catenin (Ser33/37/Thr41) or total endogenous cytosolic β-catenin. Total cellular β-catenin levels did not change.

The TOPflash luciferase reporter (TOP) contains six TCF/LEF-1 binding sites and is a commonly used measure of Wnt pathway mediated TCF/LEF-1 transcriptional activity [2], [3], [4], [10], [12], [26], [28], [29], [36], [38]. Because it is primarily cytosolic (Fig. 1a, 2, and Fig. S1), it is speculated exogenous LRP6-ICD indirectly enhances TOP transcription through amplification of cytosolic β-catenin. However, it is also possible that TCF/LEF-1 activity can be modulated by nuclear LRP6-ICD. To gain a better understanding how localization affects LRP6-ICD's ability to modulate TCF/LEF-1 activity, the TOP assay was carried out on HEK 293T cells transiently transfected with LRP6-ICD (non-targeted) or LRP6-ICD possessing a nuclear export signal (NES) or nuclear localization signal (NLS). Nuclear fractionation of the transfected cells (Fig. 1a) show LRP6-ICD is primarily localized to the cytosol whereas ∼80% of the LRP6-ICD-NLS construct localizes to the nucleus. As expected, LRP6-ICD-NES is exclusively cytosolic. In agreement with published results [35], [36], [37], [38], [39], LRP6-ICD amplified endogenous cytosolic β-catenin levels by decreasing its phosphorylation (Fig. 3d), and significantly amplified TOP transcription by ∼30% (Fig. 3a). Similar to LRP6-ICD [35], [36], [37], [38], [39], cytosolic LRP6-ICD (i.e., LRP6-ICD-NES) stabilized endogenous cytosolic β-catenin (Fig. 3d) and enhanced TOP transcription by ∼40% (Fig. 3b). No significant difference in β-catenin stabilization was seen between LRP6-ICD and LRP6-ICD-NES which supports the observation that LRP6-ICD is primarily cytosolic. Unexpectedly, nuclear targeted LRP6-ICD (i.e. LRP6-ICD-NLS) repressed TOP transcription by ∼38% (Fig. 3c), which suggest nuclear LRP6-ICD functions as a negative TCF/LEF-1 modulatory protein. In accord with previous findings [35] and demonstrated by exogenous LRP6-ICD×5 m (which cannot be phosphorylated by GSK3β), cytosolic LRP6-ICD functions independent of GSK3β phosphorylation (Figs. 3a, b and d). Here we also show that nuclear LRP6-ICD does not have to be phosphorylated by GSK3β to negatively modulate TCF/LEF-1 activity (Fig. 3c). It should be noted that the TOP data can not be compared between LRP6-ICD, LRP6-ICD-NES and/or LRP6-ICD-NLS as the empty NES (pcDNA-NES) and NLS (EV-GFP-NLS) vectors activated TOP activity compared to lacZ or eGFP (see Materials and Methods). Modulation of TCF/LEF-1 activity was specific as none of the expression constructs significantly affected the mutant TCF/LEF-1 luciferase reporter (FOPflash; FOP) or the transfection control reporter (TK-*Renilla*; data not shown). The LRP6-ICD-NES results confirm the premise that cytosolic LRP6-ICD indirectly modulates TCF/LEF-1 activity through β-catenin. Additionally, the TOP data suggest exogenous LRP6-ICD, depending on its subcellular localization (i.e. cytosolic or nuclear), can differentially function within the Wnt pathway by positively or negatively modulating TCF/LEF-1 activity.

### Nuclear AES positively modulates TCF/LEF-1 dependent transcription

**Figure 4:**
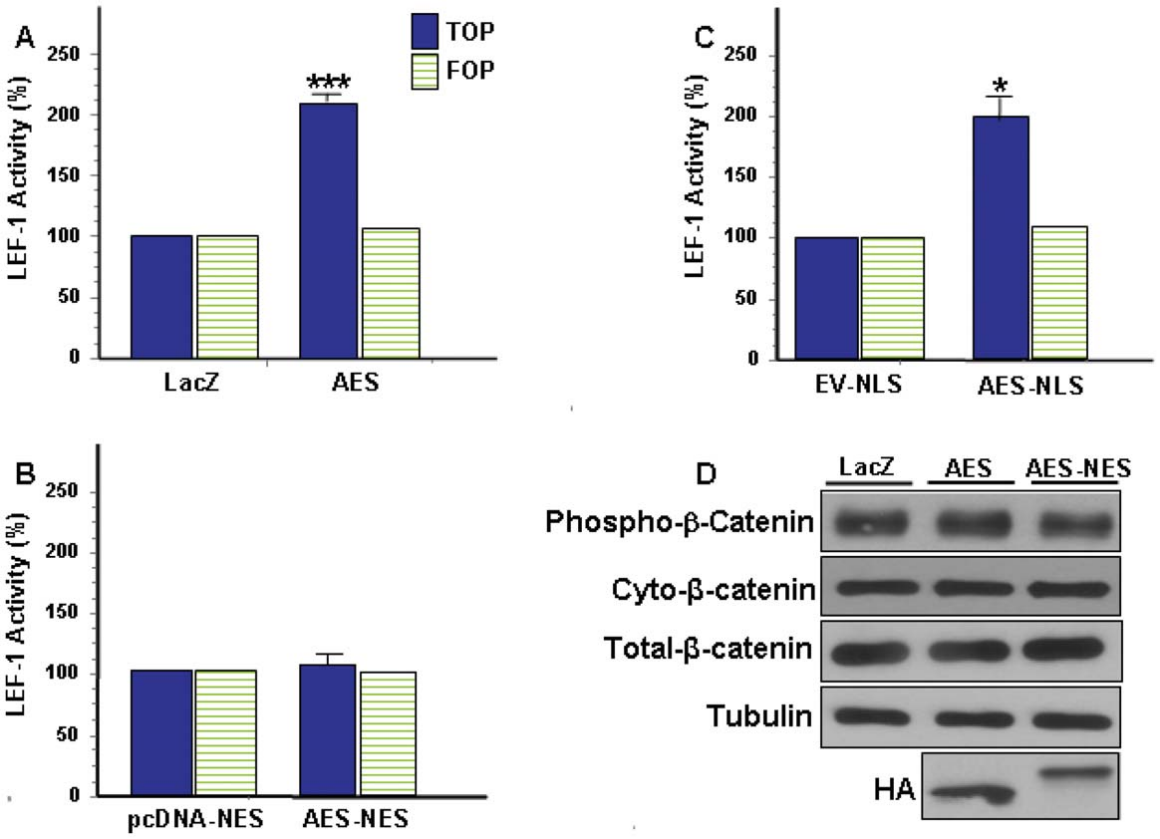
AES mediates activation of TCF/LEF-1 in a subcellular localization-dependent manner. HEK 293T cells were transfected with luciferase assay reporter constructs along with (A) control LacZ or AES-HA (AES) for 24 hrs (B) control pcDNA-NES or AES-HA-NES (AES-NES) for 48 hrs (C) control EV-NLS or AES-myc-NLS (AES-NLS) for 48 hrs and TCF/LEF-1 activity was measured. TCF/LEF-1 activity for the AES constructs is expressed as a percentage of the relevant control used in each condition: (*P<0.01, ***P<0.0001). (D) HEK 293T cells were transfected with control LacZ, AES or AES-NES. Twenty-four hrs post-transfection, cells were collected and cytosolic fractions were probed for endogenous cytosolic phospho-β-catenin (Ser33/37/Thr41) or total endogenous cytosolic β-catenin. Total cellular β-catenin levels did not change.

AES is a ubiquitously expressed ∼25 kDa cytosolic and/or nuclear protein [10], [12], [14], [17], [22]. Although AES lacks a putative NLS [11], [15] its subcellular distribution is cell type and possibly context dependent [12], [14], [17], [22], [46]. Fractionation of HEK 293T cells expressing a non-targeted AES-HA construct (i.e. AES) demonstrates AES is a nucleocytoplasmic protein that exhibits a high cytosol:nuclear localization ratio (Fig. 1b and Fig. S1). It has been shown xenopus and mouse AES (∼90% and 99% protein sequence identity to human AES) can enhance TCF/LEF-1 dependent TOP transcription [10], [12] by antagonizing the repressor function of long Groucho/TLE members [10], [11], [12], [15], [17], [18], [20], [23]. Figure 4a confirms AES acts as a positive modulator of the Wnt pathway as transient transfection of AES amplified TCF/LEF-1 dependent TOP transcription by ∼105%. It is speculated that AES functionally modulates transcription in the nucleus, but AES has also been shown to function in the cytosol [22]. To determine if AES mediated TOP transcription is spatially regulated, the TOP assay was utilized in HEK 293T cells transiently transfected with NES or NLS tagged AES constructs. Figure 1b shows the NES or NLS tagged AES constructs localize almost exclusively to the cytosol or nucleus. Whereas cytosolic AES (i.e. AES-NES) does not affect TOP transcription (Fig. 4b), nuclear targeted AES (i.e. AES-NLS) significantly enhances TOP transcription (Fig. 4c). Transcriptional modulation of TOP was specific for TCF/LEF-1 as none of the expression constructs significantly affected FOP or the transfection control reporter (TK-*Renilla*; data not shown). Neither AES nor cytosolic AES affected stabilization of endogenous cytosolic β-catenin (Fig. 4d) which was not unexpected since AES does not interact with β-catenin [8]. The results clearly show AES positively modulates TCF/LEF-1 activity and that AES functional activity is spatially regulated in HEK 293T cells. Similar to LRP6-ICD, AES subcellular distribution was not significantly altered by co-expression with LEF-1 and/or transcriptional activation of the Wnt pathway by LiCl [7] (data not shown).

### LRP6-ICD interacts with AES in the nucleus

**Figure 5:**
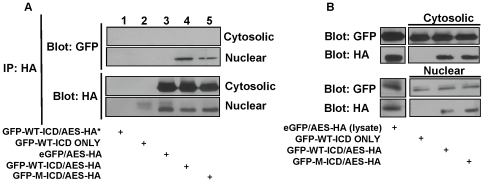
LRP6-ICD associates with AES in the nucleus. HEK 293T cells were co-transfected with GFP-LRP6-ICD, GFP-LRP6-ICD×5 m or eGFP-C1 along with AES-HA. Forty-eight hrs post-transfection cells were separated into cytosolic and nuclear fractions. (A) Cytosolic and nuclear fraction were immunoprecipitated with an anti-HA antibody against AES-HA and the presence of the GFP tagged LRP6-ICD constructs was detected in the immunoprecipitate with an anti-GFP antibody. The immunoblot was then reprobed with an anti-HA antibody. The first three lanes represent controls for non-specific interaction between the anti-HA antibody, beads and/or constructs. Note: (*) refers to control lysate that was incubated with non-immune mouse IgG1 antibody to prevent non-specific binding. (B) Blots showing expression of indicated constructs for each sample in the cytosolic and nuclear fraction (5 µg/fraction).

Previously we reported the LRP6-ICD interacted with AES in a yeast 2 hybrid assay [39] and AES is moderately expressed in HEK 293T cells [10], [22]. Therefore, interaction with a nuclear protein that positively modulates TCF/LEF-1 activity, such as AES (Fig. 4c), is a possible mechanism by which nuclear targeted LRP6-ICD negatively modulates TCF/LEF-1 activity (Fig. 3c). To determine a possible interaction, HEK 293T cells were co-transfected with AES and LRP6-ICD, fractionated and a co-immunoprecipitation assay (CoIP) was carried out on the cytosolic and nuclear fractions. The CoIP confirms the yeast 2 hybrid data that LRP6-ICD interacts with AES *in situ* (Fig. 5a) and that the interaction is exclusively nuclear (Fig. 5a; GFP blot, lane 4). The data also shows the AES/LRP6-ICD interaction does not require GSK3β mediated phosphorylation of LRP6-ICD as the nuclear phospho-mutant LRP6-ICD×5 m interacted with AES (Fig. 5a; GFP blot, lane 5). The control samples confirm the absence of a non-specific interaction between the constructs, anti-HA antibody and/or magnetic beads (Fig. 5a; GFP blot, lanes 1–3).

### Nuclear LRP6-ICD represses AES mediated activation of TCF/LEF-1 dependent transcription

**Figure 6:**
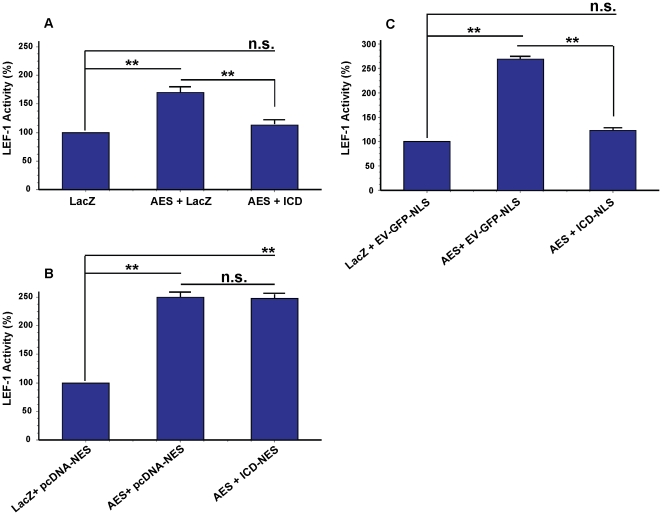
Repression of AES mediated TCF/LEF-1 transcription by nuclear LRP6-ICD. HEK 293T cells were transfected with luciferase assay reporter constructs (TOP reporter) along with (**A**) control LacZ, control LacZ and AES-HA (AES) or AES-HA and GFP-LRP6-ICD (ICD) for 24 hrs (**B**) control LacZ and control pcDNA-NES, control pcDNA-NES and AES-HA or AES-HA and GFP-LRP6-ICD-NES (ICD-NES) for 48 hrs (**C**) control LacZ and control EV-GFP-NLS, control EV-GFP-NLS and AES-HA or AES-HA and GFP-LRP6-ICD-NLS (ICD-NLS) for 24 hrs and TCF/LEF-1 activity was measured. TCF/LEF-1 activity is expressed as a percentage of the relevant control(s) used in each condition: (**P<0.001).

By interacting with AES in the nucleus, it is possible that nuclear targeted LRP6-ICD repressed TCF/LEF-1 dependent TOP transcription (Fig. 3d) by repressing AES mediated transcription (Fig. 4a). To determine this, TCF/LEF-1 activity was measured in HEK 293T cells co-transfected with AES along with LRP6-ICD, LRP6-ICD-NES or LRP6-ICD-NLS. As shown above, AES overexpression induced TOP transcription (Fig. 4a) but co-expression with LRP6-ICD, which exhibits nuclear localization, significantly repressed AES mediated TOP transcription by ∼65% (Fig. 6a). When LRP6-ICD was excluded from the nucleus, AES transcriptional activity was not repressed (Fig. 6b), but repression was re-established when LRP6-ICD was targeted to the nucleus (∼137.6%; Fig. 6c). Repression of AES mediated TOP transcription by nuclear targeted LRP6-ICD and not cytosolic LRP6-ICD combined with the CoIP/interaction data (Fig. 5a) demonstrate LRP6-ICD and AES functionally interact exclusively in the nucleus.

## Discussion

The Wnt signaling pathway is a dynamic and complex transcriptional regulatory pathway whose activation or repression influences the expression of nearly 2,000 genes affecting cell processes from proliferation to differentiation [1], [3], [5]. Robust activation of the pathway is required to initiate expression of Wnt target genes [8], [9], but similar to other pathways [47], [48], [49], [50], transcriptional activity of the pathway can be positively or negatively modulated by various extracellular, cytosolic and/or nuclear proteins [5], [6], [10], [26], [28], [30], [31], [32], [33], [35], [36], [51]. Therefore, transcriptional activity of the Wnt pathway does not exclusively exist in an “on” or “off” state but can range from high to low activity as a method to fine tune pathway activity/output in response to the dynamic cellular environment [5], [6], [10], [26], [28], [29], [30], [31], [32], [33], [34], [35], [36].

Through various protein-protein interactions and post-translational modifications, Wnt pathway transcriptional activity is commonly modulated by influencing the stability and/or activity of cytosolic/nuclear β-catenin [2], [3], [5], [52], [53], [54], [55], [56] as well as the transcriptional activity of TCF/LEF-1 [3], [8], [9], [10], [13], [30], [31]. In this study, we report a novel modulatory function for the soluble LRP6 intracellular domain (LRP6-ICD), the proteolytic product of LRP6, in that it can positively or negatively modulate Wnt pathway transcriptional activity (i.e., β-catenin stabilization and/or TCF/LEF-1 activity) depending upon its subcellular localization and differential protein-protein interactions. Cytosolic LRP6-ICD positively modulates TCF/LEF-1 activity indirectly through β-catenin. Further, we show the LRP6-ICD can translocate to the nucleus where it negatively modulates TCF/LEF-1 activity by interacting with and repressing nuclear AES, a positive TCF/LEF-1 modulatory protein [10], [12]. We also demonstrate the functional relationship between LRP6-ICD and AES is exclusively nuclear.

Cleavage, release, nuclear translocation and subsequent transcriptional modulation by the soluble intracellular domain (ICD) of a transmembrane receptor is a novel signaling mechanism that has been demonstrated for various cell surface receptors including Fz and the LRP6 family member, LRP1 [40], [41], [42], [43], [44]. As a result, such receptors (e.g., LRP -1 and -6 and Fz) can modulate transcription through multiple mechanisms. In some cases both the full length receptor and corresponding soluble ICD similarly affect transcription (e.g., Fz) [40], [44] whereas certain ICD's differentially affect transcription compared to their full length receptor (e.g., PC1, LRP1) [43], [57]. In this respect and similar to LRP1/LRP1-ICD, activated full length LRP6 positively affects transcription whereas the soluble nuclear LRP6-ICD negatively modulates TCF/LEF-1 activity.

While modulation of TCF/LEF-1 activity by LRP6-ICD or AES was significant, the scale of modulation in relation to overexpression clearly indicates that both proteins act as modulators and not primary effectors of Wnt pathway transcriptional activity. Although expression of endogenous Wnt target genes has been shown for primary activators of the Wnt pathway [2], no significant changes could be detected for LRP6-ICD or AES (data not shown). This was not unexpected as robust activation of the pathway, as shown for overexpression of primary activators, is required to initiate expression of Wnt target genes [8], [9]. It is also possible that LRP6-ICD and/or AES are capable of modulating only a specific subset of TCF/LEF-1 dependent Wnt target genes that were not included in our analysis.

Although differential modulation of transcription has been shown for non-ICD proteins, such as Hipk1 which differentially modulates β-catenin:TCF/LEF-1 transcription [32], there are no reports to our knowledge showing a particular ICD can differentially modulate transcription [40], [41], [42], [43], [44], [57], [58]. We demonstrate here for the first time [41], [42], to our knowledge, that a released ICD has dual functions as both a positive and negative modulator of transcription whose functional activity is dictated by a combination of localization and differential protein-protein interactions. Cytosolic LRP6-ICD positively modulates Wnt pathway activity by interacting with [35], [38], [39] and attenuating GSK3β [35], [37], [38], [39], a negative Wnt pathway regulator, which indirectly up-regulates TCF/LEF-1 activity through enhanced β-catenin stabilization. However, nuclear LRP6-ICD negatively modulates TCF/LEF-1 activity by interacting with AES, which functions in a dominant negative manner over the TCF/LEF-1 repressive activities of long Groucho/TLE members. By interacting with and possibly sequestering AES, nuclear LRP6-ICD may attenuate the dominant negative activity of AES, thereby allowing long Groucho/TLE members to reengage in full repression of TCF/LEF-1.

We show that the functional activity of LRP6-ICD and AES as well as their functional relationship is regulated in part by localization. Both proteins lack a putative NES or NLS and therefore regulation by the importin or exportin family of proteins seems unlikely [59]. Although the size of LRP6-ICD (∼20 kDa) and AES (∼25 kDa) allow for nuclear diffusion [60], it is reasonable to assume the transcriptional activity of the Wnt pathway is not modulated by the random diffusion of effector proteins. Subcellular distribution of LRP6-ICD does not appear to be affected by cell type as we showed it is primarily cytosolic in CHO, HEK 293T and COS (data not shown) cells which suggests LRP6-ICD functions primarily as a positive modulator within the Wnt pathway. Perhaps similar to the soluble Fz-ICD [40], LRP6-ICD nuclear localization is regulated through interaction with other proteins in a context-dependent manner. In contrast, AES subcellular distribution appears to be influenced by cell type as it is exclusively nuclear in Gonadotropin releasing neurons [17], cytosolic in COS cells [12], [14] and here we show nucleocytoplasmic distribution in HEK 293T cells. Although AES is primarily cytosolic in HEK 293T cells, we show nuclear AES functionally modulates TCF/LEF-1 dependent transcription. This suggests AES activity is also spatially regulated in HEK 293T cells. It should be noted that transcriptional activation of the Wnt pathway by LiCl [7] and/or co-expression of LEF-1 did not significantly alter the subcellular distribution of LRP6-ICD or AES in HEK 293T cells (data not shown). Therefore, we were unable to determine the regulatory mechanism(s) and/or proteins that mediate their subcellular distribution.

The assembly of transcriptionally active or repressive complexes depends on the concentrations of long Groucho/TLE and β-catenin protein(s) in the nucleus and their affinities for TCF/LEF-1 proteins [8]. This delicate balance is important as Wnt pathway transcriptional activity influences a broad range of cellular functions. For example, the balance between long Groucho/TLE members (via HDAC recruitment) and β-catenin on TCF transcriptional activity regulates oligodendrocyte differentiation, timing of differentiation and maturation [13]. Therefore, effector proteins such as AES and/or LRP6-ICD which are capable of modulating TCF/LEF-1 activity through long Groucho/TLE and/or β-catenin, have the potential to influence Wnt pathway mediated cellular functions such as proliferation/differentiation. However, the influence modulatory proteins have on cellular function can be difficult to quantify for it can be influenced by environmental cues, cell type, specific cellular function analyzed and/or the magnitude by which Wnt pathway transcriptional activity is modulated, just to name of few.

In summary, we show the soluble LRP6-ICD differentially modulates Wnt pathway transcriptional activity depending upon its subcellular localization and differential protein-protein interactions. In future studies it will be interesting to further examine how the LRP6-ICD/AES interaction affects cellular function mediated by the Wnt pathway and the cellular cues that regulate their functional localization.

## Supporting Information

Figure S1 Subcellular distribution of LRP6-ICD and AES in HEK 293T cells. HEK 293T cells were transfected with eGFP (top row), GFP-LRP6-ICD (middle row) or AES-HA (bottom row) for 48hrs and stained with anti-GFP or anti-HA antibody and DAPI as previously described.
(0.60 MB TIF)

## References

[1] Vlad A, Rohrs S, Klein-Hitpass L, Muller O (2008). The first five years of the Wnt targetome.. Cell Signal.

[2] MacDonald BT, Yokota C, Tamai K, Zeng X, He X (2008). Wnt signal amplification via activity, cooperativity, and regulation of multiple intracellular PPPSP motifs in the Wnt co-receptor LRP6.. J Biol Chem.

[3] He X, Semenov M, Tamai K, Zeng X (2004). LDL receptor-related proteins 5 and 6 in Wnt/beta-catenin signaling: arrows point the way.. Development.

[4] Wolf J, Palmby TR, Gavard J, Williams BO, Gutkind JS (2008). Multiple PPPS/TP motifs act in a combinatorial fashion to transduce Wnt signaling through LRP6.. FEBS Lett.

[5] Sierra J, Yoshida T, Joazeiro CA, Jones KA (2006). The APC tumor suppressor counteracts beta-catenin activation and H3K4 methylation at Wnt target genes.. Genes Dev.

[6] Caricasole A, Copani A, Caraci F, Aronica E, Rozemuller AJ (2004). Induction of Dickkopf-1, a negative modulator of the Wnt pathway, is associated with neuronal degeneration in Alzheimer's brain.. J Neurosci.

[7] Zeng X, Tamai K, Doble B, Li S, Huang H (2005). A dual-kinase mechanism for Wnt co-receptor phosphorylation and activation.. Nature.

[8] Daniels DL, Weis WI (2005). Beta-catenin directly displaces Groucho/TLE repressors from Tcf/Lef in Wnt-mediated transcription activation.. Nat Struct Mol Biol.

[9] Cinnamon E, Paroush Z (2008). Context-dependent regulation of Groucho/TLE-mediated repression.. Curr Opin Genet Dev.

[10] Brantjes H, Roose J, van De Wetering M, Clevers H (2001). All Tcf HMG box transcription factors interact with Groucho-related co-repressors.. Nucleic Acids Res.

[11] Chen G, Courey AJ (2000). Groucho/TLE family proteins and transcriptional repression.. Gene.

[12] Roose J, Molenaar M, Peterson J, Hurenkamp J, Brantjes H (1998). The Xenopus Wnt effector XTcf-3 interacts with Groucho-related transcriptional repressors.. Nature.

[13] Ye F, Chen Y, Hoang T, Montgomery RL, Zhao XH (2009). HDAC1 and HDAC2 regulate oligodendrocyte differentiation by disrupting the beta-catenin-TCF interaction.. Nat Neurosci.

[14] Cavallo RA, Cox RT, Moline MM, Roose J, Polevoy GA (1998). Drosophila Tcf and Groucho interact to repress Wingless signalling activity.. Nature.

[15] Gasperowicz M, Otto F (2005). Mammalian Groucho homologs: redundancy or specificity?. J Cell Biochem.

[16] Chen G, Fernandez J, Mische S, Courey AJ (1999). A functional interaction between the histone deacetylase Rpd3 and the corepressor groucho in Drosophila development.. Genes Dev.

[17] Rave-Harel N, Miller NL, Givens ML, Mellon PL (2005). The Groucho-related gene family regulates the gonadotropin-releasing hormone gene through interaction with the homeodomain proteins MSX1 and OCT1.. J Biol Chem.

[18] Bajoghli B (2007). Evolution of the Groucho/Tle gene family: gene organization and duplication events.. Dev Genes Evol.

[19] Chen G, Nguyen PH, Courey AJ (1998). A role for Groucho tetramerization in transcriptional repression.. Mol Cell Biol.

[20] Pinto M, Lobe CG (1996). Products of the grg (Groucho-related gene) family can dimerize through the amino-terminal Q domain.. J Biol Chem.

[21] Courey AJ, Jia S (2001). Transcriptional repression: the long and the short of it.. Genes Dev.

[22] Jan Y, Matter M, Pai JT, Chen YL, Pilch J (2004). A mitochondrial protein, Bit1, mediates apoptosis regulated by integrins and Groucho/TLE corepressors.. Cell.

[23] Mallo M, Lieberman PM, Gridley T (1995). Possible involvement of the mouse Grg protein in transcription.. Cell Mol Biol Res.

[24] Tetsuka T, Uranishi H, Imai H, Ono T, Sonta S (2000). Inhibition of nuclear factor-kappaB-mediated transcription by association with the amino-terminal enhancer of split, a Groucho-related protein lacking WD40 repeats.. J Biol Chem.

[25] Yu X, Li P, Roeder RG, Wang Z (2001). Inhibition of androgen receptor-mediated transcription by amino-terminal enhancer of split.. Mol Cell Biol.

[26] Katayama R, Ishioka T, Takada S, Takada R, Fujita N (2010). Modulation of Wnt signaling by the nuclear localization of cellular FLIP-L.. J Cell Sci.

[27] Mi K, Johnson GV (2007). Regulated proteolytic processing of LRP6 results in release of its intracellular domain.. J Neurochem.

[28] Binnerts ME, Kim KA, Bright JM, Patel SM, Tran K (2007). R-Spondin1 regulates Wnt signaling by inhibiting internalization of LRP6.. Proc Natl Acad Sci U S A.

[29] Tezuka N, Brown AM, Yanagawa S (2007). GRB10 binds to LRP6, the Wnt co-receptor and inhibits canonical Wnt signaling pathway.. Biochem Biophys Res Commun.

[30] Sampson EM, Haque ZK, Ku MC, Tevosian SG, Albanese C (2001). Negative regulation of the Wnt-beta-catenin pathway by the transcriptional repressor HBP1.. Embo J.

[31] Sachdev S, Bruhn L, Sieber H, Pichler A, Melchior F (2001). PIASy, a nuclear matrix-associated SUMO E3 ligase, represses LEF1 activity by sequestration into nuclear bodies.. Genes Dev.

[32] Louie SH, Yang XY, Conrad WH, Muster J, Angers S (2009). Modulation of the beta-catenin signaling pathway by the dishevelled-associated protein Hipk1.. PLoS ONE.

[33] Zhao J, Kim KA, Abo A (2009). Tipping the balance: modulating the Wnt pathway for tissue repair.. Trends Biotechnol.

[34] Yamamoto H, Sakane H, Michiue T, Kikuchi A (2008). Wnt3a and Dkk1 regulate distinct internalization pathways of LRP6 to tune the activation of beta-catenin signaling.. Dev Cell.

[35] Beagle B, Mi K, Johnson GV (2009). Phosphorylation of PPP(S/T)P motif of the free LRP6 intracellular domain is not required to activate the Wnt/beta-catenin pathway and attenuate GSK3beta activity.. J Cell Biochem.

[36] Mi K, Johnson GV (2005). Role of the intracellular domains of LRP5 and LRP6 in activating the Wnt canonical pathway.. J Cell Biochem.

[37] Cselenyi CS, Jernigan KK, Tahinci E, Thorne CA, Lee LA (2008). LRP6 transduces a canonical Wnt signal independently of Axin degradation by inhibiting GSK3's phosphorylation of beta-catenin.. Proc Natl Acad Sci U S A.

[38] Piao S, Lee SH, Kim H, Yum S, Stamos JL (2008). Direct inhibition of GSK3beta by the phosphorylated cytoplasmic domain of LRP6 in Wnt/beta-catenin signaling.. PLoS ONE.

[39] Mi K, Dolan PJ, Johnson GV (2006). The low density lipoprotein receptor-related protein 6 interacts with glycogen synthase kinase 3 and attenuates activity.. J Biol Chem.

[40] Ataman B, Ashley J, Gorczyca D, Gorczyca M, Mathew D (2006). Nuclear trafficking of Drosophila Frizzled-2 during synapse development requires the PDZ protein dGRIP.. Proc Natl Acad Sci U S A.

[41] Kopan R, Ilagan MX (2004). Gamma-secretase: proteasome of the membrane?. Nat Rev Mol Cell Biol.

[42] Landman N, Kim TW (2004). Got RIP? Presenilin-dependent intramembrane proteolysis in growth factor receptor signaling.. Cytokine Growth Factor Rev.

[43] Kinoshita A, Shah T, Tangredi MM, Strickland DK, Hyman BT (2003). The intracellular domain of the low density lipoprotein receptor-related protein modulates transactivation mediated by amyloid precursor protein and Fe65.. J Biol Chem.

[44] Mathew D, Ataman B, Chen J, Zhang Y, Cumberledge S (2005). Wingless signaling at synapses is through cleavage and nuclear import of receptor DFrizzled2.. Science.

[45] Milakovic T, Tucholski J, McCoy E, Johnson GV (2004). Intracellular localization and activity state of tissue transglutaminase differentially impacts cell death.. J Biol Chem.

[46] Mallo M, Gendron-Maguire M, Harbison ML, Gridley T (1995). Protein characterization and targeted disruption of Grg, a mouse gene related to the groucho transcript of the Drosophila Enhancer of split complex.. Dev Dyn.

[47] Sakai T, Aoyama M, Kusakabe T, Tsuda M, Satake H Functional diversity of signaling pathways through G protein-coupled receptor heterodimerization with a species-specific orphan receptor subtype.. Mol Biol Evol.

[48] Bardwell L (2008). Signal transduction: turning a switch into a rheostat.. Curr Biol.

[49] Sun JF, Phung T, Shiojima I, Felske T, Upalakalin JN (2005). Microvascular patterning is controlled by fine-tuning the Akt signal.. Proc Natl Acad Sci U S A.

[50] Yokomizo T, Dzierzak E (2008). Fine-tuning of hematopoietic stem cell homeostasis: novel role for ubiquitin ligase.. Genes Dev.

[51] Gordon MD, Nusse R (2006). Wnt signaling: multiple pathways, multiple receptors, and multiple transcription factors.. J Biol Chem.

[52] Cong F, Varmus H (2004). Nuclear-cytoplasmic shuttling of Axin regulates subcellular localization of beta-catenin.. Proc Natl Acad Sci U S A.

[53] Hannoush RN (2008). Kinetics of Wnt-driven beta-catenin stabilization revealed by quantitative and temporal imaging.. PLoS ONE.

[54] Cong F, Schweizer L, Varmus H (2004). Wnt signals across the plasma membrane to activate the beta-catenin pathway by forming oligomers containing its receptors, Frizzled and LRP.. Development.

[55] Gregorieff A, Clevers H (2005). Wnt signaling in the intestinal epithelium: from endoderm to cancer.. Genes Dev.

[56] Tago K, Nakamura T, Nishita M, Hyodo J, Nagai S (2000). Inhibition of Wnt signaling by ICAT, a novel beta-catenin-interacting protein.. Genes Dev.

[57] Low SH, Vasanth S, Larson CH, Mukherjee S, Sharma N (2006). Polycystin-1, STAT6, and P100 function in a pathway that transduces ciliary mechanosensation and is activated in polycystic kidney disease.. Dev Cell.

[58] Kinoshita A, Whelan CM, Smith CJ, Berezovska O, Hyman BT (2002). Direct visualization of the gamma secretase-generated carboxyl-terminal domain of the amyloid precursor protein: association with Fe65 and translocation to the nucleus.. J Neurochem.

[59] Ullman KS, Powers MA, Forbes DJ (1997). Nuclear export receptors: from importin to exportin.. Cell.

[60] Liu F, Wagner S, Campbell RB, Nickerson JA, Schiffer CA (2005). PTEN enters the nucleus by diffusion.. J Cell Biochem.

