# Supplementary figures and images for: Differential Modulation of TCF/LEF-1 Activity by the Soluble LRP6-ICD
Source: PLoS One. 2010 Jul 28;5(7):e11821. doi: 10.1371/journal.pone.0011821 (PMC2911377; doi:10.1371/journal.pone.0011821)

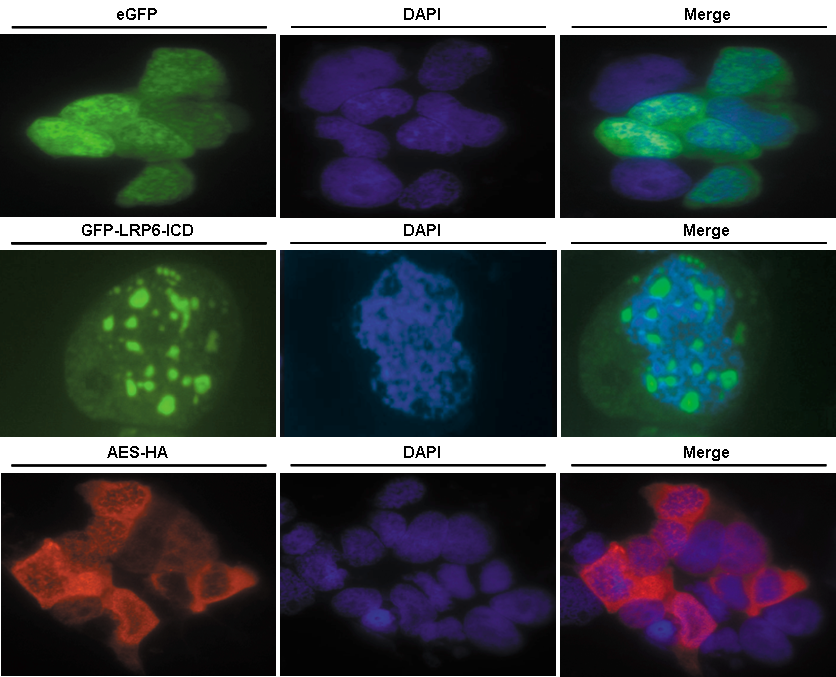

Supplement: Figure S1 — Subcellular distribution of LRP6-ICD and AES in HEK 293T cells. HEK 293T cells were transfected with eGFP (top row), GFP-LRP6-ICD (middle row) or AES-HA (bottom row) for 48hrs and stained with anti-GFP or anti-HA antibody and DAPI as previously described. (0.60 MB TIF) [file pone.0011821.s001.tif]
